# Multivariate Gaussian Copula Mutual Information to Estimate Functional Connectivity with Less Random Architecture

**DOI:** 10.3390/e24050631

**Published:** 2022-04-29

**Authors:** Mahnaz Ashrafi, Hamid Soltanian-Zadeh

**Affiliations:** Control and Intelligent Processing Center of Excellence (CIPCE), School of Electrical and Computer Engineering, University of Tehran, Tehran 1439957131, Iran; mahnaz.ashrafi@ut.ac.ir

**Keywords:** mutual information, functional connectivity, resting-state fMRI, linear correlation

## Abstract

Recognition of a brain region’s interaction is an important field in neuroscience. Most studies use the Pearson correlation to find the interaction between the regions. According to the experimental evidence, there is a nonlinear dependence between the activities of different brain regions that is ignored by Pearson correlation as a linear measure. Typically, the average activity of each region is used as input because it is a univariate measure. This dimensional reduction, i.e., averaging, leads to a loss of spatial information across voxels within the region. In this study, we propose using an information-theoretic measure, multivariate mutual information (mvMI), as a nonlinear dependence to find the interaction between regions. This measure, which has been recently proposed, simplifies the mutual information calculation complexity using the Gaussian copula. Using simulated data, we show that the using this measure overcomes the mentioned limitations. Additionally using the real resting-state fMRI data, we compare the level of significance and randomness of graphs constructed using different methods. Our results indicate that the proposed method estimates the functional connectivity more significantly and leads to a smaller number of random connections than the common measure, Pearson correlation. Moreover, we find that the similarity of the estimated functional networks of the individuals is higher when the proposed method is used.

## 1. Introduction

A brain network is defined by its nodes and edges. Different regions are considered nodes. Edges are the interactions between different regions. Each region consists of many voxels, and each voxel has a time series derived from fMRI data. There are different methods for defining the edges or interactions between different nodes. Functional connectivity (FC) is a well-known approach that produces a functional brain network. Analytically, FC is defined as the statistical interaction between the temporal activities of two distinct regions. Interactions between the regions support different cognitive processes [1]. Deviation from the FC structure of normal individuals is considered a biomarker for some diseases such as schizophrenia [2], bipolar depressive disorders [3], and attention deficit disorder [4]. Therefore, using FC as a biomarker has been widely considered recently.

Various methods have been proposed to estimate the FC between two regions. Some measures such as Granger causality (GC) and transfer entropy calculate the directed connectivity between two regions, while others such as Pearson correlation, independent component analysis (ICA), and coherence measures calculate undirectional interactions [5,6]. Moreover, some new measures such as interaction information defined based on mutual information have been introduced to estimate FC [7]. Among the undirected measures focused on in this study, Pearson correlation (PCor) is the most common approach. While the calculation of this measure is straightforward, it has some disadvantages. Recent studies have found that PCor is not sufficient to characterize the statistical dependence between two regions [8]. There are two distinct limitations to the linear correlation methods which can detect some spurious connections. Using this measure, signals from two anatomically separated brain regions may appear correlated and the regions may appear functionally connected [9,10,11,12]. However, a strong correlation between two regions may not guarantee that there is a functional connection between the underlying neurons. For instance, external or common inputs can lead to the correlation.

Linear correlation has two important disadvantages as an FC measure. First, the linear correlation is a bivariate measure that needs a single time series for each region as an input. Typically, multiple time series of voxels within each region are reduced to a single time series by averaging across voxels or by taking the first principal component. Techniques such as multivoxel pattern analysis and representation similarity analysis have shown that there are relative patterns across voxels within each region [13,14]. This reduction discards the spatial information distributed in thousands of signals within the voxels in a given region [15,16]. For instance, it has been shown that some information about mental processes and cognitive states is detected by multivariate pattern analysis, while average activity discards this information [17,18]. The second limitation of PCor is that the linear correlation cannot detect the nonlinear dependencies between brain regions. Previous studies have shown that there are nonlinear dependencies between time series during the resting state. This nonlinear analysis of fMRI signals performs better than linear correlation [19,20,21,22]. Su et al. [23] have found that considering nonlinear dependencies between fMRI signals can better discriminate schizophrenic patients from healthy subjects. Moreover, individual cognitive differences can be predicted better by taking into account the nonlinear properties of region interactions [24].

Recently, using information-theoretic quantities such as mutual information and transfer entropy has become more attractive in analyzing neuroimaging data [25]. One application of these quantities is to find the interaction between neurons [26,27] or brain regions [28]. For instance, a recent paper has proposed using a new measure, interaction information, to estimate the FC using the mutual information. Although this method takes into account the nonlinear interaction, it ignores the spatial information within voxels by taking the average across voxels [7]. Generally, an outstanding advantage of the information-theoretic quantities such as mutual information is that they do not rely on the prior assumptions about the relationship between the time series. On the other hand, linear correlation implicitly assumes a multivariate normal distribution for random variables.

In this paper, we propose using an information-theoretic measure as a functional connectivity metric that can overcome the two mentioned limitations. To this end, both nonlinear interaction and multidimensional signals have been considered. Here, we call this measure multivariate mutual information (mvMI). This measure is defined by mutual information and estimated by using the Gaussian copula notion. It is worth noting that we use the “multivariate” term as we use all voxel activities within each region to estimate the functional connectivity rather than using the average activity. Mutual information can be calculated by means of different approaches. Because of the complexity of computation and implementation, mutual information has gained less attention in the functional connectivity area. Using a copula as a statistical concept can simplify the *MI* estimation for experimental data. Moreover, we show that by using a copula, mutual information can be extended to calculate the dependence between two multidimensional variables. This property can overcome the limitation of PCor which needs univariate inputs. Here, we use Gaussian copula mutual information which was proposed by Ince and colleagues to estimate mutual information [29]. We start by presenting the theoretical background of the proposed measure, multivariate *MI* (mvMI). Then, we generate simulation data and define different scenarios to evaluate the performance of the proposed measure in the face of the mentioned limitations. The simulation results indicate that mvMI can detect nonlinear and multidimensional dependences. Moreover, it is less sensitive to additive noise. Then, we apply the proposed measure to real resting-state fMRI data. We compare the proposed measure with linear correlation in terms of significance level and randomness. We show that the mvMI-based functional network architecture is closer to the well-known topology of the brain, i.e., small-world architecture. As a complex network that has a highly efficient small-world organization, the small-world organization of the brain supports efficient information flow at low wiring and energy cost [30]. Brain dysfunctions and diseases lead to a deviation from the small-world architecture, shifting towards a random structure.

In other words, using mvMI as an estimator of FC leads to a nonrandom topology. Finally, we measure the similarity of the FC matrices obtained for different subjects using different FC measures. The results show that the similarity between subjects in the mvMI approach is larger than that in the conventional PCor approach.

## 2. Materials and Methods

### 2.1. Participants

Fifty-five young healthy right-handed individuals were recruited from the academic community and the local population living in Tehran, Iran. Participants’ age was in the range of 18–26 years (22 females and 23 males). They were university undergraduate paid volunteers. Participants completed a brief questionnaire including questions regarding medical or psychiatric disorders. Ethical approval for the study was obtained from the Iran University of Medical Sciences, Tehran, Iran.

### 2.2. MRI Data Acquisition and Preprocessing

All MRI data were acquired on a Siemens 3 Tesla scanner with a 64-channel head coil. Structural images were acquired using a magnetization-prepared rapid acquisition with gradient echo (MPRAGE) pulse sequence with the following parameters: TR/TE = 2500/3.18 ms, flip angle = 8 degrees, voxel size = 1 mm isotropic, and field of view = 244 mm. Echo-planar images sensitive to BOLD contrast were acquired using the following parameters: TR/TE = 2000/30 ms, flip angle = 80 degrees, slice thickness = 4 mm, and voxel size = 4 × 3 × 3 ${\mathrm{mm}}^{3}$. During the resting-state fMRI scan, the participants were asked to remain awake with their eyes open and not to think about anything in particular.

Functional MRI data were preprocessed using the CONN functional connectivity toolbox in MATLAB 2017b (https://web.conn-toolbox.org/ accessed on 15 January 2021) [31]. During the preprocessing steps, the functional images were slice-timing corrected, realigned, normalized (in the 2 mm Montreal Neurological Institute (MNI) space), and smoothed. The Artifact Detection Tool was used to detect outliers (>3 SD and >0.5 mm) for subsequent scrubbing regression. The structural images were segmented into gray matter, white matter (WM), and cerebral spinal fluid (CSF) and normalized to the MNI space. Then, linear regression using WM and CSF signals, linear trend, and subject motion (six rotation/translation motion parameters and six first-order temporal derivatives) was conducted to remove confounding effects. The residual blood-oxygen-level-dependent (BOLD) time series was band-pass filtered (0.01–0.1 Hz).

### 2.3. Information-Theoretic Estimation of Functional Connectivity

#### 2.3.1. Information Theory Quantities

Information theory is a mathematical framework for the quantification, storage, and communication of information. Entropy, mostly known as the Shannon entropy, is a key quantity in information theory defined as the amount of uncertainty involved in the value of a random variable. Let *X* and *Y* be two continuous random variables having marginal pdf $f_{X}$ and $f_{Y}$ respectively. Denote by $f_{X,Y}$ the joint pdf of *X* and *Y*. The Shannon entropy of variable *X* is defined as

(1) $$H\left(X\right)=-\int_{x}f_{X}\left(x\right)log_{2}f_{X}\left(x\right)dx$$

The joint entropy of *X* and *Y* is defined as

(2) $$H\left(X,Y\right)=-\int_{x,y}f_{X,Y}\left(x,y\right)logf_{X,Y}\left(x,y\right)dxdy$$

Mutual information is another fundamental quantity used to calculate the amount of information about one random variable by observing other random variables. For two discrete random variables, *X* and *Y*, mutual information is given by the following formula:

(3) $$MI\left(X,Y\right)=\int_{x}\int_{y}f_{X,Y}\left(x,y\right)log_{2}\frac{f_{X,Y}\left(x,y\right)}{f_{X}\left(x\right)f_{Y}\left(y\right)}dxdy,$$

Mutual information can be rewritten based on Shannon entropy as

(4) $$MI\left(X,Y\right)=H\left(X\right)+H\left(Y\right)-H\left(X,Y\right)$$

#### 2.3.2. Information-Theoretic Quantities of Gaussian Variables

For the multivariate Gaussian random variables, there is a closed-form solution for entropy:

(5) $$H\left(X\right)=\frac{1}{2}log_{2}[\left(2\pi e{)}^{k}\left|\Sigma_{X}\right|\right]$$

where $\Sigma_{X}$ and *k* are the covariance matrix and dimensionality of *X*, respectively. Using this closed-form expression of entropy leads to an exact definition of mutual information. Based on Equations (4) and (5), the mutual information of two random variables with Gaussian distribution can be calculated as follows [32]:

(6) $$MI_{G}\left(X,Y\right)=\frac{1}{2}log_{2}\left(\frac{\left|\Sigma_{X}\right|\left|\Sigma_{Y}\right|}{\left|\Sigma_{XY}\right|}\right)$$

where $\Sigma_{X}$ and $\Sigma_{Y}$ are the covariance matrices of *X* and *Y*, respectively, and $\Sigma_{XY}$ is the joint covariance matrix of variables *X* and *Y*. For two univariate random variables, *X* and *Y*, Equation (6) can be rewritten based on the Pearson correlation between *X* and *Y*, i.e., *Corr*(*X,Y*), as follows:

(7) $$MI_{G}\left(X,Y\right)=\frac{1}{2}log_{2}(1-Corr\left(X,Y{)}^{2}\right)$$

#### 2.3.3. Copulas

A copula is another approach for determining the dependency between random variables. The copula is a multivariate cumulative distribution function for which the marginal probability distribution of each variable is uniform in the interval [0, 1]. Sklar’s theorem states that a multivariate cumulative distribution function (CDF), $F_{1,\ldots ,k}\left(x_{1},\ldots ,x_{k}\right)=Pr\left(X_{1}\leq x_{1},\ldots ,X_{k}\leq x_{k}\right)$, can be defined by its marginal CDF, $F_{i}\left(x_{i}\right)=Pr\left(X_{i}\leq x_{i}\right)i=1,\ldots ,k$, and a copula *C*:

(8) $$F_{1,\ldots ,k}\left(x_{1},\ldots ,x_{k}\right)=C\left(F_{1}\left(x_{1}\right),\ldots ,F_{k}\left(x_{k}\right)\right)$$

The theorem indicates that the copula is unique if the marginals $F_{X_{i}}$ are continuous [33]. Notably, a copula can be considered as the joint CDF of the random vector $\left(U_{1},\ldots ,U_{k}\right)$, where each element is derived using the following transformation:

(9) $$u_{1}=F_{1}\left(x_{1}\right),\ldots ,u_{k}=F_{k}\left(x_{k}\right)$$

Thus, the copula can be defined as

(10) $$C\left(u_{1},\ldots ,u_{k}\right)=F_{1,\ldots ,k}\left(x_{1},\ldots ,x_{k}\right)=F_{1,\ldots ,k}\left(F_{1}^{-1}\left(u_{1}\right),\ldots ,F_{k}^{-1}\left(u_{k}\right)\right)$$

The joint probability density function of random variables can be written as

(11) $$f_{1,\ldots ,k}\left(x_{1},\ldots ,x_{k}\right)=c\left(F_{1}\left(x_{1}\right),\ldots ,F_{k}\left(x_{k}\right)\right)\prod_{i=1}^{k}f_{k}\left(x_{k}\right)$$

where $c\left(u_{1},\ldots ,u_{k}\right)=\frac{\partial^{k}C\left(u_{1},\ldots ,u_{k}\right)}{\partial u_{1}\ldots \partial u_{k}}$ is the copula density. Using Equation (3), mutual information can be rewritten as follows:

(12) $$\begin{array}{c} MI\left(X_{1},\ldots ,X_{k}\right)=\\ \int \left[c\left(F_{1}\left(x_{1}\right),\ldots ,F_{k}\left(x_{k}\right)\right)\prod_{i=1}^{k}f_{k}\left(x_{k}\right))\right]logc\left(F_{1}\left(x_{1}\right),\ldots ,F_{k}\left(x_{k}\right)\right)dx_{1}\ldots dx_{k} \end{array}\;=\int c\left(u_{1},\ldots ,u_{k}\right)log\left(c\left(u_{1},\ldots ,u_{k}\right)\right)du_{1}\ldots du_{k}\;=-H\left(c\left(u_{1},\ldots ,u_{k}\right)\right)$$

Thus, the *MI* is equivalent to the negative of copula entropy. *MI* between two random variables *X* and *Y* with transformation $u=F_{X}\left(x\right),v=F_{Y}\left(y\right)$ can be obtained as

(13) $$MI\left(X,Y\right)=-H\left(c\left(u,v\right)\right)$$

Thus, the *MI* between two random variables can be obtained independently of the marginal distribution of the variables. For more details about the copula and its properties, see [34].

#### 2.3.4. Estimating *MI* Using Gaussian Copula

Calculating the mutual information through (3) is computationally complex. In other words, as estimation of the joint probability density function (pdf) of non-Gaussian distributed data is hard, the estimation of mutual information is also difficult. Thus, little attention has been paid to this method as an estimator of FC. In the previous section, we illustrated that copulas offer a natural approach for estimating mutual information, independently of the marginal distributions. As the copula entropy and thus the *MI* do not depend on the marginal distributions, without loss of generality, the marginals can be transformed to the standard Gaussian distributions. Since the copula is mostly unknown, Robin Ince and colleagues used the Gaussian approximation [29]. Here, we use the Gaussian copula as an estimation for the unknown copula.

The Gaussian copula entropy for two random variables *X* and *Y* can be derived as follows [32]:

(14) $$MI\left(X,Y\right)=H\left(c_{G}\right)=-\frac{1}{2}log_{2}\left(1-r^{2}\right)$$

where *r* is the correlation between the transformed Gaussian random variables. It is obvious that if the real copula is not Gaussian, the *MI* derived using (14) is not accurate. Since for a given mean and covariance matrix, the joint Gaussian distribution has the maximum entropy [35], the Gaussian copula also has the maximum entropy. As *MI* is the negative copula entropy, the Gaussian copula provides a lower bound for the true *MI* [36].

#### 2.3.5. Multivariate Mutual Information in Neuroimaging

FC is defined as the statistical dependence between each pair of brain regions. Each region consists of many voxels, and each voxel has a time series that is derived from the fMRI data. Most studies summarize each region’s activity in a single time series obtained by taking the average across voxels. This dimensional reduction from the voxel dimension to a one-dimensional signal leads to the loss of spatial information between voxels. The main reason for this dimension reduction is that most FC quantities such as Pearson correlation’s inputs should be one-dimensional. Mutual information as a dependence quantity has the capability to estimate the dependence between two multidimensional random variables. In the previous section, we showed that *MI* can be calculated by means of the copula concept which is independent of the marginal distribution (Equation (12)). For a given covariance matrix Σ, the Gaussian copula can be written as

(15) $$C\left(u\right)=\Phi_{\Sigma }\left(\Phi^{-1}\left(u_{1}\right),\ldots ,\Phi^{-1}\left(u_{d}\right)\right))$$

where $\Phi^{-1}$ is the inverse cumulative distribution function of a standard normal distribution and $\Phi_{\Sigma }$ is the joint cumulative distribution function of a multivariate normal distribution with zero mean and correlation matrix equal to Σ. The density function can be written as

(16) $$c\left(u_{1},\ldots ,u_{k}\right)=\frac{1}{\sqrt{det\Sigma }}exp(-\frac{1}{2}\left[\Phi^{-1}\left(u_{1}\right),\ldots ,\Phi^{-1}\left(u_{k}\right)\right]\left(\Sigma^{-1}-I\right)\left[\Phi^{-1}\left(u_{1}\right),\ldots ,\Phi^{-1}\left(u_{k}\right){]}^{T}\right)$$

Equation (4) is the entropy of a multivariate normal distribution. We can use this equation to find the entropy of the Gaussian copula.

Here, to find the interaction between two regions which are multidimensional variables, we transform the marginal distribution of the variables to standard normal distribution. This transformation is performed since the *MI* is independent of the marginal distribution. Then, we use (4) to find the copula entropy of transformed variables, which is equal to *MI* based on (12).

### 2.4. Functional Connectivity Measures

Linear correlation methods such as Pearson correlation are the simplest approaches for calculating FC. For two time series, *x* and *y* with n time points, the Pearson correlation is defined as follows:

(17) $$Pcor=\frac{\sum_{i=1}^{n}\left(x_{i}-\bar{x}\right)\left(y_{i}-\bar{y}\right)}{\sqrt{\sum_{i=1}^{n}{\left(x_{i}-\bar{x}\right)}^{2}}\sqrt{\sum_{i=1}^{n}{\left(y_{i}-\bar{y}\right)}^{2}}}$$

where $\bar{x}$ and $\bar{y}$ are the mean values of *x* and *y*, respectively. This measure can only detect the linear dependence between two univariate time series. Each region contains many signals related to different voxels. Before calculating the correlation between two regions, it is necessary to reduce each region’s activation to a single time series. Taking the average across voxels within each region is the most common method for this dimension reduction in functional connectivity studies. The Pearson correlation which uses the average signal is called PCor. If the voxel activities in a region are homogeneous, this approach is the best choice. However, as the homogeneity in the regions decreases, an average time series is not a good representative of the regional activity. Singular value decomposition is an alternative method, whose advantage arises when the regions are not homogeneous. As the second measure of FC, SVD, we summarize each region’s activity using the temporal singular vector corresponding to the largest singular value of the regional activity. *MI* is a more general measure. As a multivariate estimator, one important feature of *MI* is that it is capable of finding the statistical dependence between two multidimensional variables. Thus, for calculating the interaction between two regions with multiple voxels, it is not necessary to reduce the voxel time series within each region to a single time series, e.g., the average time series.

Another advantage of *MI* over PCor is that it is a nonlinear dependence estimator. To pinpoint which aspect of mvMI leads to a difference from PCor, we use both univariate and multivariate versions of *MI* in our investigation. For the univariate version, which we name uvMI, we use *MI* to estimate the association between the average time series of two brain regions. For the multivariate version, named mvMI, we use multiple time series from each region to quantify the FC between two regions. To calculate the mvMI, we start with a principal component analysis over each region’s time series and select the first 5 principal components of each region. Indeed, by applying principal component analysis (PCA) to each region’s time series, we select the most important components as a representation of that region’s activities. Then, FC between two regions is estimated by calculating the *MI* between the 5 selected principal components of the regions.

Unlike the PCor, which has a value within the range of −1 to 1, the mutual information’s value is more open-ended and can range from 0 for complete independence to infinity for complete dependence. To have a fair comparison, we rescale both *MI* measures to the [0, 1] range using a power transformation. As the *MI* is a positive measure, the absolute values of PCor have been used throughout this article.

### 2.5. Simulation Design

We propose using *MI* as an FC metric because of two advantages of *MI* over Pearson correlation, the capabilities of detecting nonlinear and multidimensional dependence. In the previous section, a computationally efficient approximation of *MI* was presented. To evaluate if the approximation preserves the considered advantages, we use simulated data. We compare the performance of *MI*-based quantities and Pearson correlation through different dependencies between two simulated regions.

We simulate the time series of two distinct regions containing 100 and 150 voxels with 500 time points. The time series of the first region is generated using a multivariate normal distribution. The time series of the second region is calculated from those of the first region using a given mapping function *f*. This function controls the interaction between the two regions. It can be a nonlinear function such as a power function. By changing *f*, the performance of *MI* for linear and nonlinear interactions can be evaluated. To evaluate the performance of different measures by the simulated data, we estimate the null distribution of each measure by calculating the FC value between the regions of the null data, which is obtained by shuffling the time points randomly for each voxel. Then, we calculate the distance between each FC value and the 95th percentile of the null distribution. This distance is considered as the performance quantity.

#### Simulation Scenarios

In order to compare the performance of the mvMI measure with Pearson, four different simulated scenarios have been defined. In each scenario, we consider four different measures of FC, two Pearson correlation-based measures, and the two mutual information-based measures. Through different scenarios, we assess the performance of the proposed measure (mvMI) from different aspects. In each scenario, a different mapping function f is defined to generate the time series in the second region, based on the first region’s time series. Let *X_t_* and *Y_t_* be two vectors containing the *N_x_* and *N_y_* values which represent the voxel activation within two regions at time point *t*. For each scenario, the second region’s activation, *Y_t_*, is defined as a function of the first one, *X_t_*:

(18) $$Y_{t}=f\left(X_{t},T\right)$$

where *T* is the mapping matrix and *f* is the function that determines the relation between the two regions. For example, for a linear voxel-to-voxel mapping from region 1 to region 2, *f* is a multiplication function and *T* is an *N_x_* × *N_y_* mapping matrix in which *N_x_* × *N_x_* elements are an identity matrix and the remaining elements are just random noise An independent Gaussian noise is added to both *X_t_* and *Y_t_* as the measurement noise, $w_{t}$:

(19) $$Y_{t}=X_{t}\times T+w_{t}$$

For instance, to compare the performance of different measures in detecting nonlinear interaction, we use the elementwise power function as a nonlinear function. Indeed, the second region’s activity at time *t* $Y_{t}$ is derived as

(20) $$Y_{t}={\left(X_{t}\times T\right)}^{2}+w_{t}$$

**Figure 1:**
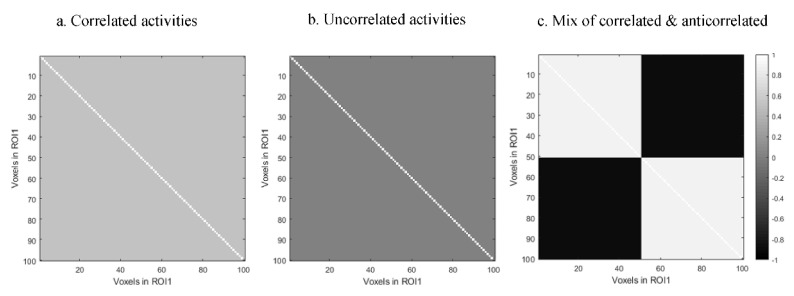
Different covariance matrices used to simulate different levels of homogeneity within the first region: (**a**) correlated activities, i.e., constant matrix; (**b**) uncorrelated activities obtained using an identity matrix; (**c**) mixtures of correlated and anticorrelated activities within the first region. These covariance matrices are used in generating the first region’s activities using a multivariate normal distribution.

The homogeneity of voxels in a region is an important property that directly affects the average time series. In the situation where all voxels within a region have the same activation, reducing the ROI activation to a single time series does not lead to any loss of information. As the homogeneity of the ROI activation decreases, a greater amount of information will be lost by taking the average. We use the covariance matrix as a parameter to control the first region’s homogeneity level. In each scenario, we consider three different covariance matrices for the first region’s time series which demonstrates the homogeneity level of that region. We consider two extreme covariance matrices, constant and identity. We use the constant matrix to generate homogeneous activity, i.e., the time series are highly positively correlated. For identity one, the time series within each region will be uncorrelated or independent. One middle condition is taken into account which is constituted of two constant positive and negative values which indicate positively correlated and anticorrelated, respectively. These covariance matrices are illustrated in Figure 1. Through these scenarios, the performances of different FC measures, PCor, SVD uvMI, and mvMI, are examined. Nonlinearity, multivariate dependencies, and sensitivity to the structured noise are the main properties that are investigated through these scenarios.

### 2.6. Real Data Analysis

The brain contains functional communities called resting-state networks. These networks show high-level within-community functional interaction and low-level interaction strengths between communities. The connections can be divided into within- and between-network categories. If the two nodes linked by the connection are located in the same network, the connection is considered a within-network connection; otherwise, the connection is considered a between-network connection. At first, we compare the functional brain networks derived using different estimators by calculating the correlation between different functional connectivity matrices for within and between connections.

Using different approaches, we compare the performance of the proposed estimator (mvMI) with the other measures. For each connection and estimator, it is examined whether the connections are significant or not. By shuffling the time points of voxel time series within each region, the null distribution is obtained. By comparing the true value of each connection with the 95th percentile threshold of the null distribution, the connection is characterized as significant or insignificant. If the true value is greater than the 95th percentile level, it will be considered a significant connection.

As a property of a normal functional network, the randomness level of each measure’s connectivity matrix is measured. Previous studies have illustrated that the brain network has a nonrandom pattern. Deviation from this pattern leads to known neuropathologies [37]. For each connectivity measure, the nonrandomness level is determined using the Networkx, a graph theory package (https://networkx.org/ accessed on 15 December 2021). The main idea is to quantify how close a connectivity matrix is to a random matrix containing random elements. There is a baseline for functional brain networks which is dominated by common resting-state networks across participants. Indeed, recent studies have indicated that resting-state functional brain networks share certain similar patterns including the connectivity weights and the spatial distribution of resting-state networks [38,39]. For instance, the default mode network (DMN) and the frontoparietal network are two well-known networks thought to be activated during the resting state. These networks do not vary significantly across different healthy subjects [40]. To investigate the similarity of functional networks between different subjects, the correlation between each subject’s connectivity matrix and the average connectivity matrix is computed.

## 3. Results

In this section, the results of the comparison of the proposed estimator (mvMI) with the most common measure (PCor) on both the simulated and real rs-fMRI data are provided.

### 3.1. Simulation Results

In this section, we present the simulation results for four different scenarios. It is worth noting that for all scenarios we use the simulation data and covariance matrices explained in Section 2.5. The first four scenarios differ in *f* and *T* definitions, while they have the same covariance matrices. In the last scenario, the robustness of each measure to noise is evaluated. In all scenarios, we obtain FC 100 times to avoid random results. Illustrated results are the average performance across 100 repetitions.

#### 3.1.1. Linear Interaction between Two Regions

**Figure 2:**
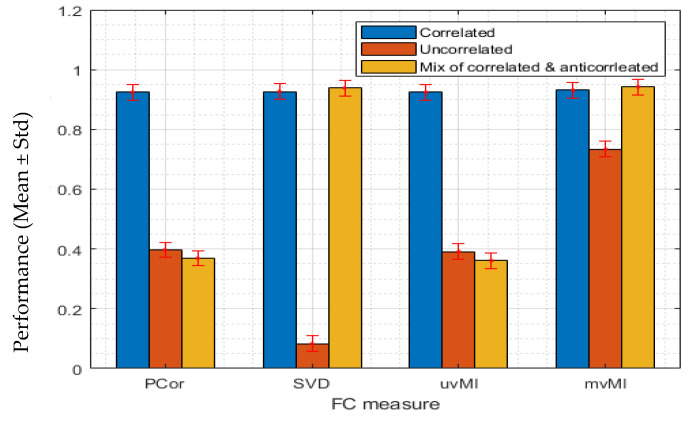
We simulate a one-to-one voxel mapping which shows a linear interaction between two regions. Each bar value shows the average performance of detecting the linear interaction across 100 repetitions by using four different FC measures, PCor, SVD, uvMI, and mvMI. Error bars show the standard deviation of the performance across repetitions. The performance is defined as the distance between the FC value derived using each measure and the 95th percentile of the null distribution. The first region’s activities are generated by a multivariate normal distribution with three different covariance matrices shown in Figure 1. For each FC measure, the obtained performance using different covariance matrices is illustrated through different colors. Blue bars are related to the covariance matrix in Figure 1a which simulates the homogeneous or correlated activities within the first region. Inhomogeneous or uncorrelated activity results generated by the covariance matrix in Figure 1b are shown in orange color. Yellow bars are the result of using the covariance matrix in Figure 1c which simulates both correlated and anticorrelated activities within the first region. mvMI can detect the linear interaction for all covariance matrices better than the other measures. For linear interaction, uvMI performs as well as PCor. SVD has a weak performance in detecting interaction when activities within one region are anticorrelated.

In this section, we report the performance of different measures in detecting the linear interaction between two simulated brain regions. To implement this scenario, we generate the first region’s time series using a multivariate normal distribution with zero mean and different covariance matrices; for more details, see Section 2.5. Using Equation (19) the second region time series are generated. In this example, *T* is an *N_x_* × *N_y_* matrix whose first *N_x_* × *N_x_* elements are an identity matrix that simulates the linear interaction between two regions and whose other elements are random noise. Figure 2 shows the performance of different measures. Each color is related to a special covariance matrix. As expected, all measures can detect the linear interaction with the positively correlated activities (blue bars).

For the uncorrelated activities, mvMI outperforms the others, while SVD has the worst performance. Regardless of the covariance matrix, PCor and uvMI have the same performance, since both use the average regional activity. This result confirms that taking the average, which is used in PCor and uvMI, is a good representative of a region when all activities are correlated (blue bars). However, for the nonhomogeneous activity (orange and yellow bars), taking the average leads to performance reduction.

#### 3.1.2. Nonlinear Interaction between Two Regions

**Figure 3:**
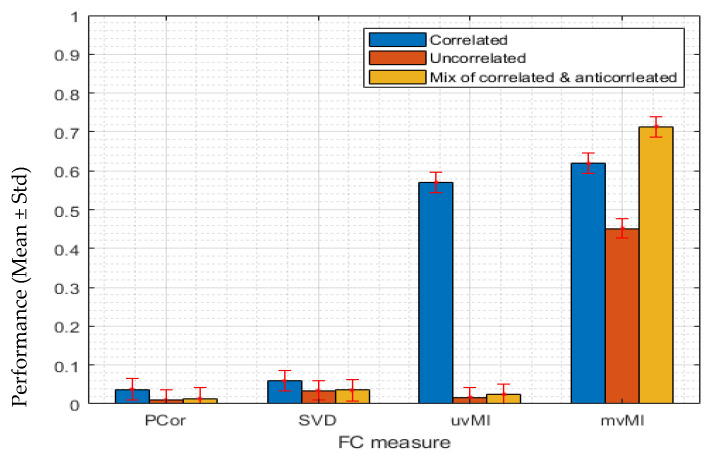
We simulate a nonlinear interaction between two regions. Each bar shows the performance of each FC measure PCor, SVD, uvMI, and mvMI in detecting the nonlinear interaction. Error bars correspond to the standard deviation of the performance across all repetitions. Performance is defined as the distance between the FC value derived using each measure and the 95th percentile of the null distribution. To simulate the nonlinear interaction, the activities within the second region are derived via a second power function of the first region’s activities. For each FC measure, we use three covariance matrices in Figure 1 to generate the first region’s activities. Performances obtained by using different covariance matrices are represented in different colors. The result of using homogeneous or correlated activities (using Figure 1a covariance matrix) and inhomogeneous or uncorrelated activities (using covariance matrix in Figure 1b) are shown by blue and orange bars, respectively. The performance related to the third covariance matrix (Figure 1c), which contains two constant values, positive and negative, has been illustrated by yellow bars. This one simulates a condition where some voxels within each region are positively correlated while the others are anticorrelated, i.e., negatively correlated. Each bar shows the mean performance of each measure across 100 repetitions. Pcor and SVD as two linear measures fail to detect the nonlinear interaction. uvMI as a measure that uses the average activity within each region only detects the nonlinear interaction in the homogeneous condition. The mvMI correctly detects this interaction using different covariance matrices.

In this scenario, we evaluate the nonlinear capability of all measures. To simulate this condition, we use a power function. Data for the second region are generated using Equation (20). In this scenario, we use the same matrix for *T* as in the previous scenario and a different power function as *f*. We again consider three different covariance matrices. Regardless of the homogeneity of the activities, i.e., covariance matrices, mvMI has the highest performance and detects the nonlinear interaction between two regions. However, uvMI can detect the nonlinear interaction only for the positively correlated condition (blue bar). While *MI* is a nonlinear dependency, detecting the nonlinear FC between two regions by uvMI is dependent on the homogeneity of the activities. As we expected, both PCor and SVD as linear measures fail to detect the nonlinear interaction (see Figure 3).

#### 3.1.3. Multivariate Interaction between Two Regions

**Figure 4:**
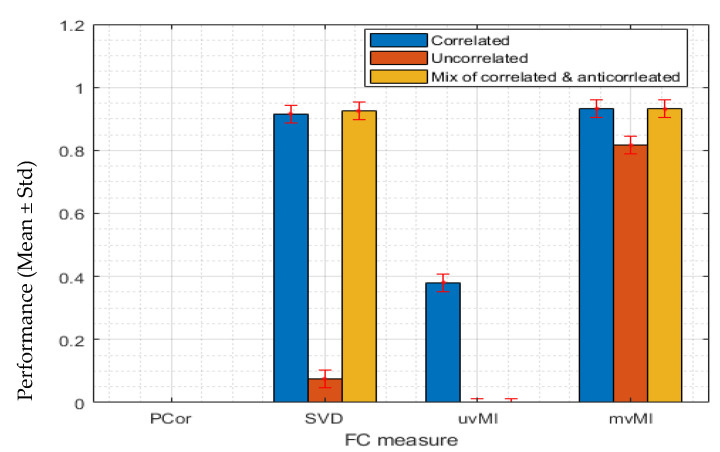
Simulation results of the performance of four different connectivity measures, PCor, SVD, uvMI, and mvMI, in detecting a multivariate interaction. Each bar shows the mean performance of each measure across 100 repetitions. Error bars correspond to the standard deviation of the performance across the different repetitions. Performance is defined as the distance between the value derived using each FC measure and the 95th percentile of the null distribution. Multivariate interaction is simulated by using a transformation matrix whose elements are derived using the multivariate normal distribution shown in panel a. The second region’s activities are derived by multiplying the first region’s activities by this transform matrix. The first region’s activities are generated using a multivariate normal distribution with three different covariance matrices in Figure 1, which are shown in three colors. Blue represents the homogeneous or correlated activities within the first region. Orange bars are related to the result of using inhomogeneous or uncorrelated activities. The third covariance matrix, which contains two constant values, positive and negative, has been illustrated in yellow. This one simulates a mixed condition of correlated and anticorrelated activities. PCor, as a measure that uses the average activity of each region, does not detect this connection. mvMI, as a multivariate measure independent of the regional homogeneity, detects this multivariate connection.

Here, we simulate a multivariate dependence between two regions using a multivariate normal distribution. In other words, *f* and *T* in Equation (20) are the multiplication function and an *N_x_* × *N_y_* matrix whose elements are generated by a multivariate normal distribution, respectively. Based on Figure 4, mvMI finds the multivariate interaction in all three conditions, while both PCor and uvMI, which are univariate measures that use average regional activities, cannot detect the multivariate interaction. SVD recognizes the interaction for positively correlated and mixed covariance matrices. This measure fails to detect the interaction when the activities are independent (orange bar).

#### 3.1.4. Additive Structural Noise

**Figure 5:**
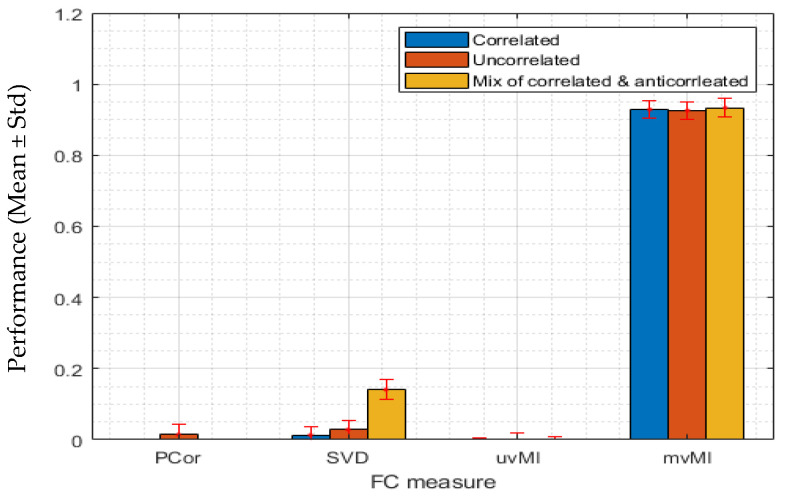
Performance of four different connectivity measures, PCor, SVD, uvMI, and mvMI, in detecting a linear interaction while the second region contains an additional same noise across all voxels named structural noise. Performance is defined as the distance between the FC value derived using each measure and the 95th percentile of the null distribution. Each bar illustrates the average performance across repetitions of each measure in detecting the FC interaction. Standard deviation of performance across repetitions is represented by the error bar on top of each bar. The first region’s activities are generated using a multivariate normal distribution with three different covariance matrices with different levels of homogeneity shown in different colors. Blue and orange are related to the homogeneous and inhomogeneous activities within the first region, respectively. The yellow bars are related to an intermediate condition, in which some voxels are positively correlated while the others are anticorrelated. All measures except mvMI are sensitive to the noise and cannot detect the linear interaction.

In this scenario, additional noise is added to the time series of the second region. The noise power is the same for all voxels. Here we simulate a linear interaction with three different covariance matrices similar to previous scenarios. This kind of noise can be present in the real data due to various reasons such as subject movement or changes in alertness across different time points. Figure 5 illustrates that additional noise has the least effect on mvMI, i.e., mvMI is more robust to noise than the other measures. The other three measures, especially PCor and SVD, cannot detect the interaction.

### 3.2. Real Data Results

#### 3.2.1. Comparing the Connectivity Matrices Derived Using Different Measures

**Figure 6:**
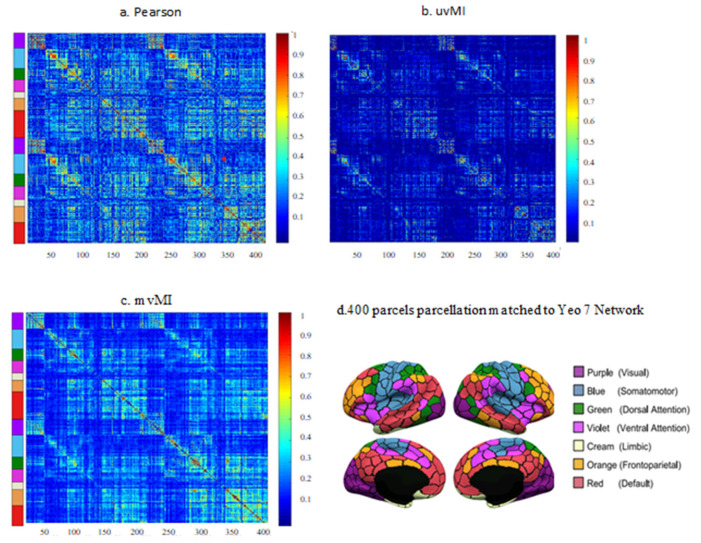
Functional connectivity matrices derived using different estimators (**a**–**c**) for a subject’s fMRI data parcellated by the 400-ROI Schaefer parcellation. (**d**) Visualization of the 400-parcel parcellation in fslr32k space; parcels were colored to match Yeo 7-network parcellation.

The connectivity matrices obtained for the 400-ROI Schaefer parcellation [41] of one participant using PCor, uvMI, and mvMI are shown in Figure 6. The ROIs are ordered according to seven resting-state functional networks similar to Yeo atlas networks [42]. Between-network connections are sparser for the mutual information measures, uvMI and mvMI, than PCor. Moreover, uvMI estimated some weak connections within networks. Since the correlation between PCor and SVD is very high, more than 0.9 for all participants, the SVD measure is excluded from real data analysis.

**Figure 7:**
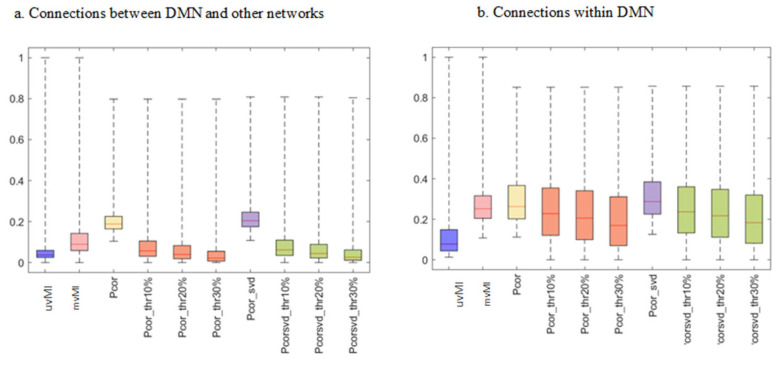
Average DMN FCs across subjects estimated by different measures, uvMI, mvMI, PCor, and SVD. Moreover, thresholded PCor and SVD with different threshold levels including 10%, 20%, and 30% of the maximum values of FCs are added to the results. Average DMN FC illustration is separated for within- and between-network connections. (**a**) Connections that connect a region within the DMN with a node in other networks (**b**). Connections whose endpoint nodes are within the DMN. PCor and SVD have the highest median values as they have more spurious connections. Thresholding removes some DMN connections and leads to smaller boxes that represent the less diverse connection values.

To further analyze network-based FC structures revealed by different measures, the connection weights of the average matrices across participants are displayed in Figure 7, where within- and between-network connections for the default mode network have been separated. There are some spurious connections in correlation-based connectivity matrices [43]. Thresholding is a common approach for cleaning these connections. However, thresholding also removes some real connections. Here, thresholding has removed some between- and within-network connections of the default mode network (DMN), which leads to a reduction in median value. The DMN is a set of regions that exhibit greater activity during the resting state [10]. Some diseases such as schizophrenia (SZ) and autism spectrum disorder (ASD) lead to reduced interactions within the DMN [40,44].

**Figure 8:**
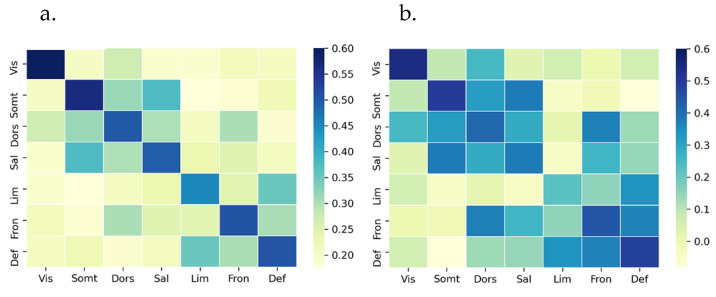
Similarity between FCs derived using mvMI with uvMI and PCor for different networks. Similarity is separated for different networks. Diagonal squares illustrate the FC measures’ similarity of connections whose connected nodes are on the same networks. Other squares represent the similarity between different measures considering the connections that connect two nodes of different networks. Similarity is calculated using the rank correlation. (**a**) Similarity between mvMI and uvMI; (**b**) similarity between mvMI and Pearson. Darker squares indicate more similarity between two related FC measures. mvMI and uvMI are more similar for within-network connections. PCor and mvMI are less similar in the visual network, while they are more similar in the DMN.

To quantify the general similarity of different measures, we compute the rank correlation between each pair of connectivity matrices generated by different measures. The average rank correlation coefficients, as a similarity metric, across all participants between mvMI and PCor and between mvMI and uvMI are 0.24 and 0.35, respectively. These values indicate that FC obtained from mvMI is more different from the PCor measure than from the uvMI measure. To better recognize the similarity of measures within each network separately, we calculate the correlation between each pair of measures for connections within each network. Figure 8 shows the average correlation across all repetitions for mvMI with uvMI and PCor. The similarity between mvMI and PCor is higher for connections that connect the nodes inside the networks, except for the limbic network. The similarity between mvMI and PCor for visual and limbic networks is less than the others.

#### 3.2.2. Comparing Insignificant Connections and Nonrandom Architecture of Functional Networks Obtained Using Different Measures

In the previous section, it has been demonstrated that the connectivity matrix obtained using mvMI is different from PCor and uvMI across some connections, especially for visual and limbic networks. In this section, the significance of the connections is investigated. First, the null distribution of each connection is derived using the permutation test. For each connection, we randomly shuffle the time points of the time series within regions connected by the given connection 100 times. The connections are divided into significant and insignificant categories according to comparing the true value of the connection with the 95th percentile value of the null distribution.

**Figure 9:**
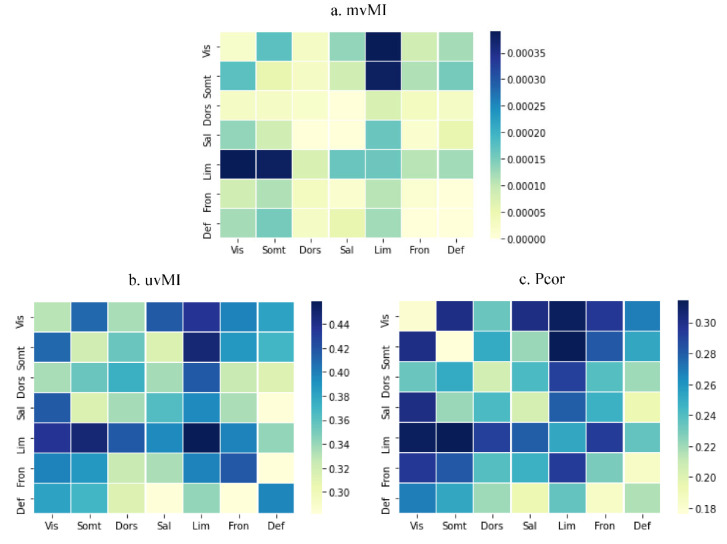
Average number of insignificant FCs across all participants for different FC measures. The result is separated for different networks. Diagonal squares show the within-network connections while the others are related to the connections that connect two nodes in different networks. Significance is determined by comparing each FC value with the 95th percentile of the null distribution obtained using the permutation test. mvMI has the lowest number of insignificant connections in comparison with other measures.

Figure 9 illustrates the average number of insignificant connections. The connection weights have significant values when using mvMI. Comparing Figure 8b and Figure 9c shows that for the PCor measure, cells with insignificant values (blue colored) in Figure 9c are those which are less similar to mvMI (yellow colored) in Figure 8b. Therefore, the insignificant connections obtained using PCor are those that are different from mvMI. This means that mvMI makes a difference in functional connections whose PCor values are insignificant. As the FC architecture of a healthy individual has a nonrandom structure that supports different cognitive functions, we evaluate the randomness level of the proposed method, mvMI. For each participant and FC measure, the sum of nonrandomness values of all edges within the functional network is calculated. The nonrandomness of an edge tends to be small when the two nodes linked by that edge are from two different communities [45]. The values for mvMI, uvMI, and PCor are 336.6, 70.07, and 27.68, respectively. The results denote that the mvMI connectivity matrix has a smaller number of random connections compared to the other two measures.

#### 3.2.3. Functional Network Similarity between Subjects

**Figure 10:**
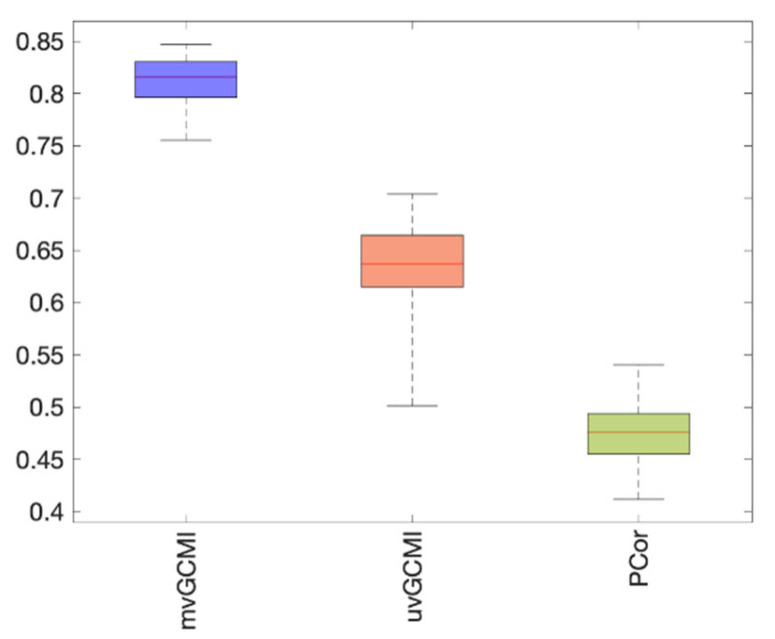
Between-subject similarity of FC matrices derived using different FC estimators, uvMI, mvMI, and PCor. Similarity between subjects is defined as the correlation between each subject’s FC matrix and the average FC matrix across subjects. Each subject correlation value is a point of the box. Each box is related to an FC estimator. FC matrices estimated by mvMI are more similar across different subjects. Using PCor leads to less similarity between subjects.

As the average time series of each region is related to the homogeneity of voxel activation within that region, the representative time series of each ROI may be different for different subjects. This leads to less similarity between different subjects’ functional networks obtained using PCor. Figure 10 shows that the similarity of the functional network between subjects for mvMI is higher than that for uvMI and PCor. As the similarity index of uvMI is larger than that of PCor, it can be concluded that both nonlinear and multivariate properties of mvMI lead to an increase in similarity between subjects.

**Figure 11:**
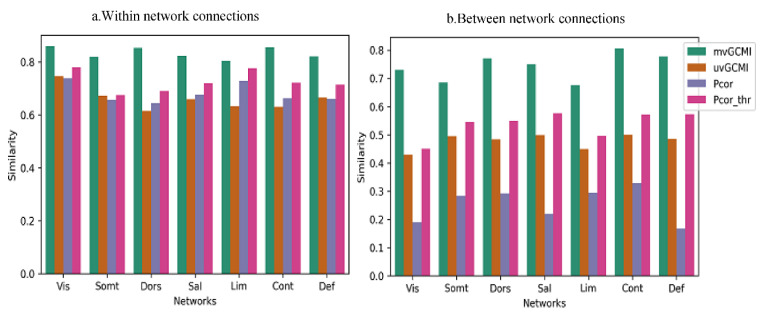
Between-subject similarity separated for within- and between-network connections. For each subject, the similarity is obtained using the correlation between the subject’s FC matrix and the average FC matrix across all subjects. Each bar illustrates the average similarity values across different subjects. (**a**) The result for connections that connect two regions located in the same network, or within-network connections. (**b**) The similarity between subjects for connections that connect two regions of different networks, called between-network connections. Different measures are separated by different colors. Each group of bars is related to a special network. Between-network connections derived using mvMI are more similar across different subjects than the other FC measures. Using PCor as a measure of FC leads to less similarity between different subjects for between-network connections.

Next, we examined the similarity for different resting-state networks in Figure 11, both within and between networks. For PCor, the connectivity structure within networks is more similar across subjects than between-network connections. In the previous section, it has been illustrated that between-network connections have a more random organization than the within-network connections. This randomness yields less similarity between subjects. To test the effect of thresholding on the between-participant similarity value, we have added thresholded PCor to our investigation. Although thresholding has improved the similarity for between-network connections, it is still less than the mvMI similarity value for all networks. PCor has the lowest similarity value for the visual and default mode networks.

## 4. Discussion

In summary, we investigated multivariate Gaussian copula mutual information as an estimator of FC. This estimator can be used as an alternative to the widely used measure PCor, which has two important limitations. These limitations motivated us to use mvMI, which is a more robust estimator than the linear correlation methods [46]. As a linear measure, PCor misses nonlinear dependences. Another limitation of PCor is that it can only utilize the univariate time series as inputs. Consequently, the time series within each region should be reduced to a single time series using methods such as averaging. This dimension reduction causes a loss of voxel-level spatial information. Using simulated data, we compared the performance of mvMI with PCor from different aspects and showed that, in contrast to PCor, mvMI detects both linear and nonlinear interactions. Moreover, in situations where the ROIs have inhomogeneous activity, we showed that mvMI detected connectivity ignored by PCor. In addition, the sensitivity of FC measures to additive Gaussian noise was examined. The results illustrated less noise sensitivity of mvMI compared to the other measures.

We started our investigation on real data by comparing the connectivity matrices derived using different FC estimators. We evaluated the similarity between mvMI and PCor across different resting-state networks. The connections were divided into within- and between-network connections. All FC measures were similar in estimating within-network connections. For between-network connections, they behaved differently, and mvMI outperformed the others, especially for the visual and limbic networks. To better recognize which features of mvMI lead to this capability, we included the univariate version of *MI* (uvMI) in our investigations.

We verified the significance of each connection by comparing the FC value with the 95th percentile of the null distribution. We found that for between-network connections in which mvMI and PCor are considerably different, PCor connections were insignificant. For example, across visual and limbic networks, PCor was less similar to mvMI. Most insignificant connections of PCor were in these networks (see Figure 8b and Figure 9c). As an example, the similarity between PCor and mvMI for connections between the DMN and the frontoparietal network was high, and the number of insignificant PCor connections between these networks was small. In other words, the number of insignificant connections of PCor was directly correlated with its similarity with mvMI. The insignificant connections were those that were more different from mvMI. One approach to remove the insignificant PCor-based connections between networks is thresholding. In our investigation, we analyzed the performance of thresholding with mvGCM. Thresholding removed the insignificant connections but also removed some significant connections. Actually, some weak connections may be important in explaining the cognitive differences of individuals. For example, it has been declared that IQ variance is mostly explained by moderately weak, long-distance connections, with only a smaller contribution of stronger connections [46]. Thus, thresholding methods may destroy some useful information.

According to previous studies, a functional brain network has a nonrandom structure with a specific architecture that supports different cognition functions [47]. For example, the brain has a small-world topology of short path length and high clustering. Deviation from this nonrandom topology is considered a biomarker of some diseases. It has been shown that there is an alteration in the small-world organization of the functional network of the brain of some patients. In other words, there is a deviation toward a random structure [48]. In addition, there is an association between the randomness level and ADHD disorder, where there is an increase in randomness during childhood and early adulthood [49]. To better understand these disorders, characterization of the nonrandomness of brain connectivity has gained attention in recent years [34]. Therefore, we assessed whether the proposed method generated a random structure or not. We found that the nonrandomness level of mvMI is greater than that of the other measures.

The resting-state networks, especially the DMN, are consistent across different subjects. We measured the similarity of functional networks across subjects. We found that the similarity of the connections estimated for different subjects using mvMI was the highest for both within- and between-network connections and was approximately 0.8. This result is consistent with that of a recent paper which has declared that the shared pattern of functional networks across different subjects generates an intersubject similarity of 0.822 ± 0.061 [38].

## Data Availability

The fMRI data used in this study are available on request from the corresponding author. The data are not publicly available due to the nature of the research in which the data were acquired where the participants did not agree for their data to be shared publicly.
